# Spatial normalization improves the quality of genotype calling for Affymetrix SNP 6.0 arrays

**DOI:** 10.1186/1471-2105-11-356

**Published:** 2010-06-29

**Authors:** High Seng Chai, Terry M Therneau, Kent R Bailey, Jean-Pierre A Kocher

**Affiliations:** 1 Division of Biomedical Statistics and Informatics, Mayo Clinic College of Medicine, Rochester, MN 55905, USA

## Abstract

**Background:**

Microarray measurements are susceptible to a variety of experimental artifacts, some of which give rise to systematic biases that are spatially dependent in a unique way on each chip. It is likely that such artifacts affect many SNP arrays, but the normalization methods used in currently available genotyping algorithms make no attempt at spatial bias correction. Here, we propose an effective single-chip spatial bias removal procedure for Affymetrix 6.0 SNP arrays or platforms with similar design features. This procedure deals with both extreme and subtle biases and is intended to be applied before standard genotype calling algorithms.

**Results:**

Application of the spatial bias adjustments on HapMap samples resulted in higher genotype call rates with equal or even better accuracy for thousands of SNPs. Consequently the normalization procedure is expected to lead to more meaningful biological inferences and could be valuable for genome-wide SNP analysis.

**Conclusions:**

Spatial normalization can potentially rescue thousands of SNPs in a genetic study at the small cost of computational time. The approach is implemented in R and available from the authors upon request.

## Background

Single nucleotide polymorphism (SNP) genotyping arrays of continually increasing resolution allow unprecedented levels of genetic information to be captured in a single experiment. They enable the identification, on a genome-wide scale, of genetic markers that may be associated with various phenotypic traits, such as disease status and drug response. However, a genotyping experiment [1,2] is a sophisticated and time consuming undertaking with many potential sources of systematic biases that are unrelated to the biological phenomena under study. These biases include variations introduced by chip manufacturing, DNA sample processing, as well as experimental conditions. Such unwanted effects can induce inflated dropped-call rates and/or genotyping errors, which in turn can compromise the statistical power of detecting association and, worse still, lead to the generation of incorrect biological hypotheses.

One form of systematic biases pertains to regional inhomogeneity of intensity measurements over the surface of a single array. These location dependent biases were first noted in two-channel microarray experiments [3,4]. Various kinds of non-biological spatial artifacts have been reported by the user community since then [5-7], including fibers, droplets and scratches that render measured intensity values useless, as well as uneven washing and temperature gradient that have a more subtle effect on intensity signals. Importantly, many of them are expected to be present in fair abundance regardless of microarray platforms [3,5,6,8,9]. The normalization methods used in the current genotype calling algorithms do not specifically address such spatial biases even though these undoubtedly contaminate experimental data and might hamper subsequent analyses. Older generations of Affymetrix SNP arrays use 20 perfect match (PM) probe pairs scattered across the chip to interrogate each SNP, so that spatial biases may be somehow mitigated by simple averaging. However, to make room for more SNPs, Affymetrix 6.0 chips reduce the number of PM probe pairs per SNP to as few as 3 (scattered across the chip) and so are more susceptible to errors induced by these biases.

Although not originally developed for the Affymetrix SNP 6.0 arrays, many techniques can be adapted to reveal the most flagrant physical defects [5,8,10]. These techniques compare the intensity value of each location in an array to the corresponding signals aggregated across a set of replicated chips or a large collection of similar type of chips. Regional accumulation of probe-wise outliers indicates the presence of physical artifacts. However, the approach is indirect and not capable of unveiling subtle to moderate spatial patterns and may overlook biases that are consistent across the chips.

In this publication, we report the development of an iterative method, designed to isolate both extreme (consequent upon debris, scratches, etc.) and subtle (indicative of uneven liquid flow rate, temperature gradient, etc.) spatial biases from actual biological signals within individual microarrays. Our spatial bias removal procedure takes advantage of the technical replicate probes embedded in the Affymetrix 6.0 chips but is equally applicable to other experimental platforms with similar feature, for instance the Illumina BeadArrays. The procedure is independent of the downstream genotype calling algorithm. We focus on assessing the effect of spatial bias on genotype calls in this manuscript although the Affymetrix 6.0 arrays can also be used to determine genomic copy number variation.

## Methods

### The design of Affymetrix SNP 6.0 array

The latest Affymetrix SNP Array 6.0 chip is a 2680 × 2572 oligonucleotide microarray, capable of simultaneously interrogating more than 900 K SNPs as well as over 900 K non-polymorphic (NP) loci that are dedicated for the detection of chromosomal copy-number variation across the human genome [11]. SNPs and NP sites are represented by clusters of identical 25-nucleotides oligomers immobilized at specific location on the microarray. A cluster of oligomers are commonly referred to as a probe. Each probe is manufactured to perfectly match nucleic acid sequences containing the corresponding SNP or NP locus. Because only two alleles are observed in nature for most SNPs, several pairs of SNP probes are used to examine a specific SNP. Probes in a probe pair differ by just one nucleotide in the position that corresponds to the SNP locus. Probes targeting the same SNPs or NP sites constitute a probeset.

Our approach leverages two key features of the design of the array. First, in contrast to some previous generations of Affymetrix SNP chips in which probes in a probeset may have different orientation (forward or reverse DNA strand) and offset (SNP resides at the centre or shifted by -4 to +4 base pairs), probe pairs in the non-control SNP probesets of the Affymetrix 6.0 arrays are strict technical replicates. In other words, all probes of a particular SNP allele have the same nucleotide sequences and should therefore exhibit identical hybridization characteristics. These probes are deposited on the chip in either triplicate or quadruplicate. Secondly, pairs in a probeset are distantly distributed on the chip, though the members of each probe pair are located in adjacent positions. These arrangements enable one to statistically identify location-dependent biases, and separate them from real biological information and noise. Note that SNP probe pairs used for quality control purposes and probes querying NP sites are not replicated within the Affymetrix 6.0 array. Strong heterogeneity in hybridization responses makes measurements of nonreplicated probes from the same probeset not directly comparable and less useful for within-array spatial biases detection. Nonreplicated probes are, therefore, not utilized in our spatial normalization procedure.

### Datasets

We shall make use of three publicly available sets of Affymetrix 6.0 cell intensity files to train, test and validate our methods. The first set consists of 5 replicated chips for one of the HapMap Phase I + II sample [12-14]. These files will be used for demonstration and training purposes in the remainder of this manuscript. The second set includes 270 chips for all HapMap Phase I + II individuals. Cel files for both Set 1 and Set 2 were obtained from Affymetrix [15]. The last set, downloadable from the SNP Affycomp website [16,17], refers to a single 'first-pass' experiment with 96 HapMap Phase I + II samples. Sets 1 and 2 are considered to be of high quality whereas Set 3 represents 'typical' quality. Sets 2 and 3 will be utilized in the Results Section for testing and validation.

### Spatial normalization procedure

Affymetrix SNP 6.0 arrays uses fluorescent labeling to quantify the amount of genomic DNAs hybridized to each probe. The levels of fluorescent signals for each chip are stored in a separate cell intensity file (.CEL) which is used as input to the proposed algorithm. Our approach to spatial normalization relies on two assumptions: 1.) that any discrepancies in intensity values between replicates are necessarily attributable to stochastic fluctuations in conjunction with spatial biases, and 2.) that the same spatial artifact affects many probes in close vicinity in a similar fashion. For probesets with replication, we decompose the measured intensities *I*_*xy*_, or their transformation, at cell (*x*,*y*) for SNP *j *with allele *k *(*k *= 1, 2) into(1)

where *A*_*jk *_is the intensity resulting from both specific and nonspecific hybridization. Fluorescence emitted by the DNA molecules represented by the probe is specific, while signals from other DNA molecules and background are considered nonspecific. The second component *S*_*xy *_denotes the spatial bias effects. Additional sources of experimental variation are captured by the spatially uncorrelated error terms *ε*_*xy *_assumed to obey a zero mean symmetrical distribution.

Model (1) is meant to be array-specific since both allelic intensities and spatial biases are likely to vary from sample to sample (see also Section 3.1). Our single-chip estimation and correction scheme is briefly described below. The motivation and justification of each step will be detailed in the coming sub-sections.

(1) Apply a generalized logarithmic (glog) transformation on *I*_*xy*_.

(2) For each SNP allele, initialize *Â*_*jk *_with the median intensity of the replicates.

(3) Estimate *Ŝ*_*xy *_*= E(S*_*xy*_) by a two-dimensional wavelet surface fitted to *I*_*xy *_- *Â*_*jk*_.

(4) Update *Â*_*jk *_with the corresponding means of the bias-adjusted signals, i.e. *I*_*xy *_- *Ŝ*_*xy*_.

(5) Iterate steps 2 and 3 until convergence.

(6) Remove outliers, i.e. probes with extreme local biases and revise *Â*_*jk*_.

#### Glog transformation

**Figure 1:**
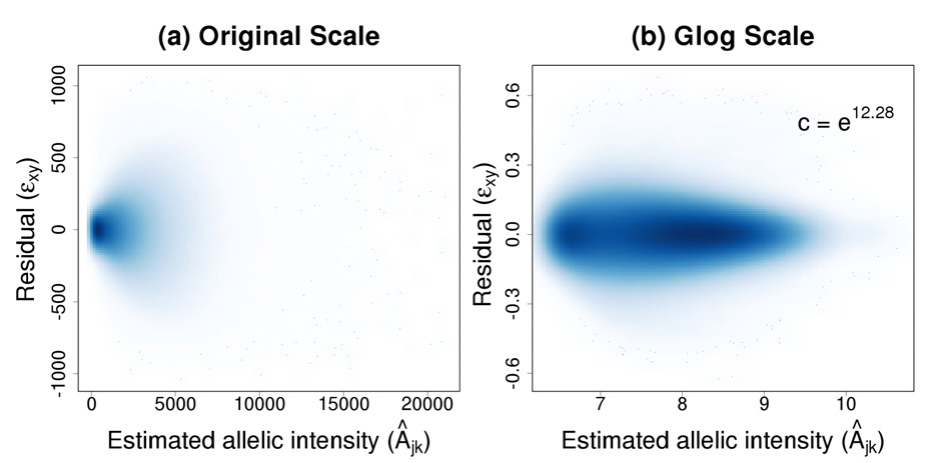
***Â*_*jk *_against ε_*xy *_scatterplots with smoothed densities color representation**. The plots correspond to the first array in Set 1 (see Method Section). Dark and light shades of blue indicate the presence of many and few points, respectively. (a) Higher *Â*_*jk *_is accompanied by larger residual variance on the raw scale. (b) Glog transformation makes *Â*_*jk *_and the variability of *ε*_*xy *_approximately independent.

As evidenced in Figure 1a, the variance of *ε*_*xy *_in Model (1) is not constant across the value of *Â*_*jk *_if the very first step above is skipped. This implies that probes with more noisy intensity levels would have a relatively larger impact on the estimation of the spatial effects. The amount of systematic information that can be borrowed across neighboring probes would also be compromised. Although the log transformation is often considered as a method for achieving constant variance, it tends to produce high variance at low regions of intensity. Glog is a family of transformations [18-20] that has been proposed for addressing the problem of heteroscedasticity that avoids this effect by bounding the logarithmic argument away from zero. Following Durbin and Rocke [21], it is defined as

We adopt the algorithm suggested by the authors for estimating *c*. In order to reduce the computational burden, we perform the estimation using only 50,000 randomly selected SNP alleles. Visual inspection of Figure 1b indicates that a satisfactorily stabilized variability is achieved across the intensity range.

#### Median or mean adjustment

Dust particles, bubbles and scratches are typical contaminants that disturb intensity signals greatly but affect only limited areas of a microarray. Hence it is reasonable to assume that most of the replicates that are constructed to be distant from each other on the chip are free from these strong perturbations. As a first approximation, we choose to calculate *Â*_*jk *_in Step 2 using the median because of its robustness against outliers. However, once the fitting of *S*_*xy *_absorbs the spatial disturbances, we conjecture that inferring *A*_*jk *_with the mean intensity value will be much more efficient since the probes are duplicated only 3 to 4 times. This motivates the use of the mean in Step 4.

#### Wavelet de-noising

**Figure 2:**
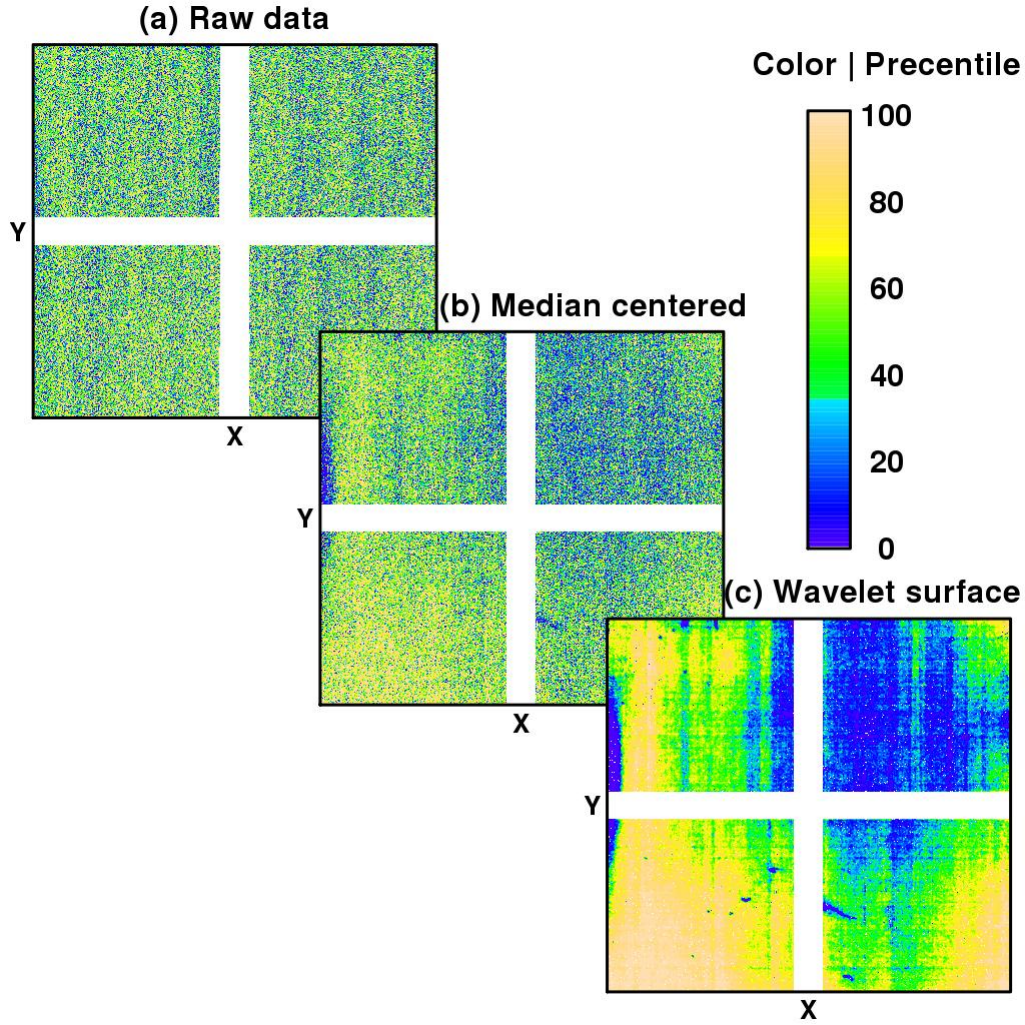
**Image plots of glog transformed intensities against their locations on the array**. The plots correspond to the first array in Set 1 (see Method Section). Colors are proportional to percentiles of the signals; white pixels correspond to non-replicated probes. (a) Raw data: no regional trend is apparent. Median (IQR) = 1150 (562, 2035). (b) Median-centered data: patterns of spatial biases become visible across the array. Median (IQR) = 0.0000 (-0.0360, 0.0362). (c) Wavelet denoising strengthens the evidence for various sources of spatial imperfection. Median (IQR) = -0.0019 (-0.0315, 0.0285). Note: wavelet surface was independently estimated from 4 regions separated by the white bands.

Step 3 intends to reconstruct the spatial biases robustly without assuming a particular structure for the underlying signal. The justifications for choosing wavelet-based methods [22] rely mainly on their theoretical properties and computational efficiency. Regional defects can show complex and discontinuous behaviors. Wavelets, a family of nonparametric techniques, are particularly suited for capturing abrupt local changes as well as smooth global trends from noisy data. We utilize maximal overlap discrete wavelet transform (MODWT) in this study. MODWT is translation invariant (x-y origin is arbitrary), can compute all coefficients in just *O*(*N *log_2 _*N*) multiplications, and does not require the number of probes along the x- or y-direction to be a power of 2. However, as with other discrete wavelet transformations, it needs a complete data array. Affymetrix 6.0 chips have 'holes' for the locations that do not contain replicated SNP probes. For example, probes allocated for alignment and control purposes or the NP probes. As a solution, we fill in these 'holes' with the average of neighboring points (see Additional File 1). A large portion of the NP probes are allocated into a horizontal and vertical stripes across the chip (see Figure 2). Rather than interpolate across these broad bands, the wavelet basis was fitted separately to each of the four corners.

The reconstruction of regional biases is performed using the 'denoise.mowdt.2d' function implemented in the R package 'waveslim', version 1.6.1. Several parameters need to be specified. We use the simple Haar wavelet and enforce the universal soft thresholding rule as a way of reducing the level of noise while preserving the significant features of the true signal. In light of the hierarchical nature of wavelet transforms, we select a decomposition level (also known as resolution level or scale) that optimized the overall reproducibility of *Â*_*jk *_across the replicated samples in our training data, i.e. Set 1. To reflect the symmetrical role played by these training arrays, we measure reproducibility in a pairwise manner through

in which *λ*_*1 *_denotes the major eigenvalue of each pairwise correlation matrix (see Additional File 2). Notice that *R*^*2 *^is simply the coefficient of determination of a simple linear Deming's model [23]: the higher the *R*^*2*^, the better the reproducibility. We set the level of decomposition to 3 as additional resolutions did not lead to a better average *R*^*2 *^value - based on 10 possible pairings from Set 1 (data not shown). Other wavelet functions were also used during the testing phase of the procedure but none gave better results.

#### An iterative procedure

Figure 2a shows the pattern (or lack thereof) of the glog transformed intensities of a particular chip. It demonstrates the point that strong differences in probe hybridization performances coupled with variation introduced by true biological signals make the raw intensities useless for detecting all but the most prominent defects. We need to estimate *A*_*jk *_first in order to isolate the effects of regional biases. Figures 2b and 2c display the *Â*_*jk *_adjusted intensities and the denoised results of applying wavelets, *Ŝ*_*xy*_, respectively. By updating in an iterative manner, each new estimations of *A*_*jk *_and *S*_*xy *_should result in a more accurate assessment of the other effects. Utilizing the replicated chips from Set 1, we observed that almost all of the improvement in average *R*^*2 *^value is achieved after only one iteration (see Additional File 3). This is perhaps not surprising since *A*_*jk *_and *S*_*xy *_are independent by design. Notice in Model (1) that adding a constant to *Â*_*jk *_would cause *Ŝ*_*xy *_to reduce by a similar amount, i.e. *Â*_*jk *_*+ Ŝ*_*xy *_= {*Â*_*jk *_*+ c*} + {*Ŝ*_*xy *_*- c*}. Thus the parameters *A*_*jk *_and *S*_*xy *_are not 'identifiable'. One might consider imposing a constraint on *Ŝ*_*xy *_to make Model (1) identifiable. We bypass this in our applications because a shift in intensity values would have no effect on the final genotyping algorithm - specifically the quantile scaling procedure.

#### Outliers

An ideal experiment with no spatial bias would generate a zero theoretical value for all *Ŝ*_*xy*_. Non-balanced thermal conditions, uneven distribution of fluids and curvatures of chip surfaces would give rise to gradual perturbation of intensity measurements. Conversely, extreme localized deviations of *Ŝ*_*xy *_from 0 are indicative of the presence of physical artifacts such as debris, blobs and scratches. Intensities of the areas affected by the latter may retain no or very little biological information thereby making a recovery of the original signals impossible. This suggests the potential need for detecting and eliminating aberrant probe signals. This can be achieved, for example, by considering *Ŝ*_*xy *_lying *n *median absolute deviation (MAD) away from the median (of all *Ŝ*_*xy*_) as outliers. One could also refine the grouping approach by reinstating spatially isolated aberrations and discarding previously approved probes that were surrounded by ≥50% immediate outliers. The value of *n *was tested on Set 1 over the range of 2 to 10 with the increment of 1. On the basis of the reproducibility measure *R*^*2*^, we estimated that *n *= 3 is sufficient to filter out 'unreliable' probes. A more comprehensive use of the neighborhood structure might improve the grouping of outliers even further. The reader is referred to Suárez-Farinãs *et. al. *[5] for an example of this approach. *Â*_*jk *_can then be recalculated using all but these unreliable probes and serve as input for genotyping analyses. Notice that the exclusion of probes with outlying *Ŝ*_*xy *_from a certain region does not automatically lead to the elimination of the corresponding SNPs, on account of the scattered allocation of probe replicates on the array.

## Results

We illustrate the presence of chip-specific spatial bias via Set 1 and evaluate the influence of spatial bias on genotype calling through the use of Sets 2 and 3. Spatial and allelic effects are calculated individually for each array using the following parameters: Haar basis, three levels of decomposition, universal soft thresholding and one iteration. Relevant entries in the cel files are then updated with the glog-back-transformed estimates of *A*_*jk*_. Conversion of intensity data into genotype calls is done by Birdseedv2 [24], a two-dimensional clustering algorithm using a Gaussian mixture model. We use the default parameter settings of Birdseedv2 in the following applications, as would most users. Original and modified cel files are genotype-called separately. The results are compared in terms of call rate, call accuracy (calibrated against genotypes acquired from the International HapMap Project database [25] - release #22 mapped to NCBI build 36) or Mendelian error. Forasmuch as Birdseedv2 cannot handle cel files with missing intensities, the outlier removal step (Step 6) of our procedure is not implemented in these examples. So any potential alteration in genotype calls must be a direct consequence of spatial effect adjustment as opposed to the selection of 'well-behaved' probes. This provides a more appropriate basis for comparison, as removal of outlying probe intensities might create an unfair advantage for the proposed approach, by eliminating problematic SNPs from both the numerator and denominator of error rate calculations.

### Set 1: five replicated chips

**Figure 3:**
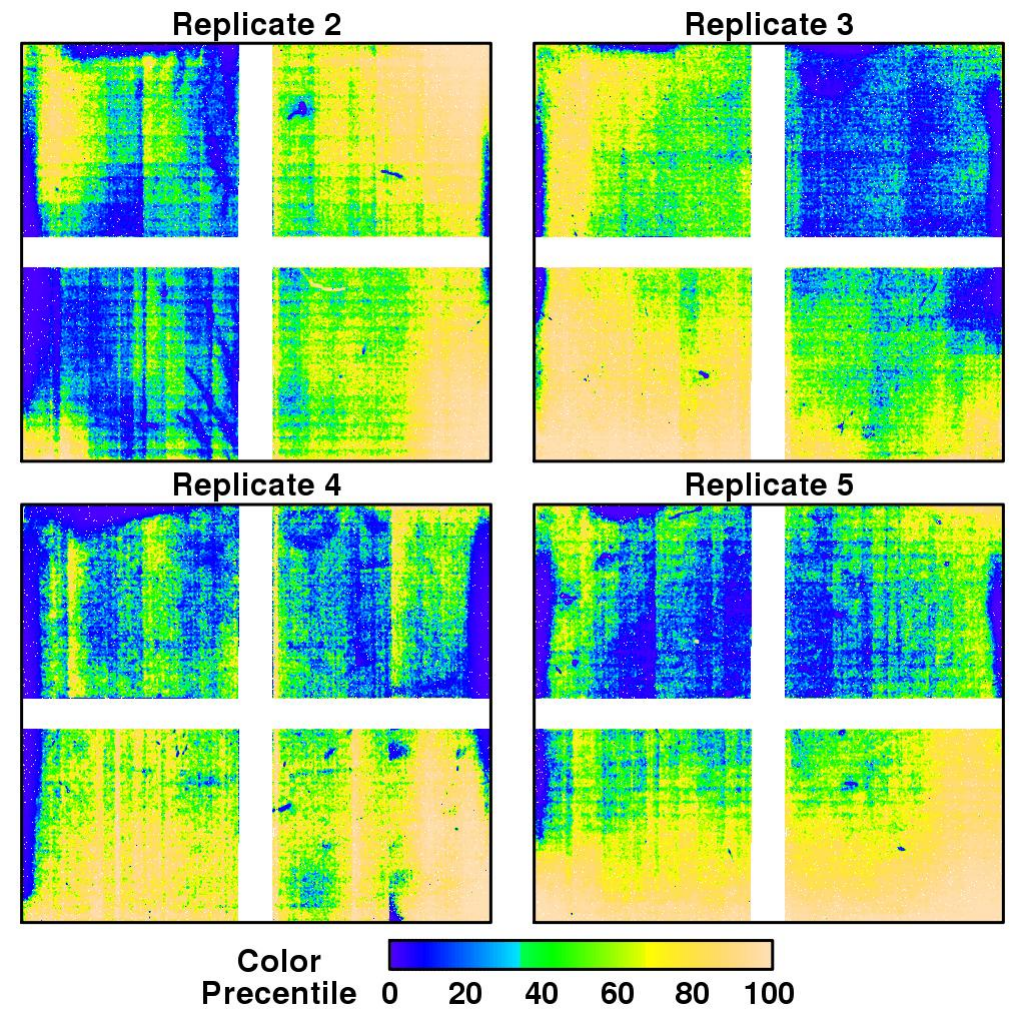
**Image plots of *Ŝ*_*xy *_for the replicated chips (Set 1)**. The image of the first array in Set 1 is shown in Figure 2c. Medians (IQR) of arrays 2-5 are -0.0001 (-0.0303, 0.0273), -0.0031 (-0.0294, 0.0279), -0.0013 (-0.0201, 0.0237) and -0.0064 (-0.0316, 0.0269), respectively.

The byproduct of our procedure, *Ŝ*_*xy*_, facilitates exploratory visualization of potential systematic spatial bias. Figure 2c and Figure 3 show *Ŝ*_*xy *_of 5 cel files arising from the same biospecimen that were treated exactly alike. The nonrandom trends suggest that all chips were affected to some extent by extreme, highly localized artifacts as well as subtle, more global biases. However, the spatial patterns are not consistent across the chips. Since the cel files were generated from the same sample, these differences must be coming from extrinsic sources independent of the true biological signals. This justifies the need of a chip-specific normalization. Although it is not the focus of this presentation, graphical illustration of *Ŝ*_*xy *_may provide new insights about the root causes of biases which, in turn, facilitate the optimization of experimental protocols. Figures 2c and 3 for instance demonstrate streaks that might indicate irregularities during the washing process.

### Set 2: 270 high-quality HapMap samples

Genotype calls for many of the SNPs in the HapMap database were independently made by two or more platforms. Concordant calls of these SNPs can be regarded as being close to the truth and, therefore, serve as a basis for calibration. We focused on SNPs that were typed by more than one non-Affymetrix platform across all 270 HapMap samples. The exclusion of Affymetrix calls would eliminate any potential platform-related bias. A strict rule of consensus was enforced: missing values were assigned to genotypes that were called differently or have at least one failed call. After filtering out SNPs with greater than 5% missing genotypes and not represented by Affymetrix SNP 6.0 arrays, a total of 96,765 SNPs (or equivalently 270 × 96,765 genotypes) were retained as benchmark for accuracy assessment. Spatial bias correction followed by the Birdseedv2 algorithm changed 34,922 genotypes in 19,602 of these SNPs as compared to running Birdseedv2 on the original cel files. Among these genotypes, the failed call rate originating from the unadjusted intensities was considerably higher (Table 1). Specifically accounting for spatial biases improved the call rate among affected genotypes from 44.0% to 67.4%. Furthermore, this improvement was achieved without inflating the number of calls discordant with the consensus HapMap calls. In other words, our method was able to restore a large fraction of the missing genotypes with good accuracy. Narrowing the results of Table 1 to 32,752 genotypes that has a 'nonmissing' consensus HapMap call (Table 2), spatial normalization converted 2,518 discordant calls into agreement with HapMap, 2,716 discordant calls into no-calls, and 16,887 no-calls into concordances. The number of calls shifting to a better category was roughly twice the number moving to a worse category in all 3 cases (all McNemar's one-tailed test p values < 0.001 in favor of using the spatial normalization, see Additional File 4).

**Table 1:** Concordance between Birdseedv2 and consensus HapMap calls of 34,922 genotypes

HapMap Call	Birdseedv2 call							
	Original				Spatial normalization			
	
	0	1	2	Missing	0	1	2	Missing
0	3356 (9.6)	2002 (5.7)	19 (0.05)	7045 (20.2)	7451 (21.3)	960 (2.7)	11 (0.03)	4000 (11.5)
1	496 (1.4)	2040 (5.8)	550 (1.6)	4257 (12.2)	393 (1.1)	4340 (12.4)	428 (1.2)	2182 (6.2)
2	17 (0.05)	2157 (6.2)	3470 (9.9)	7343 (21.0)	14 (0.04)	1135 (3.3)	7614 (21.8)	4224 (12.1)
Missing	322 (0.92)	627 (1.8)	323 (0.92)	898 (2.6)	300 (0.86)	575 (1.6)	311 (0.89)	984 (2.8)

Entries are frequencies (percentages) out of the 34,922 genotypes from Set 2 (see Method Section). Spatial bias normalization improved the call rate without inflating the number discordances with the consensus HapMap calls.

**Table 2:** Contrasting Birdseedv2 results in Table 1 using 32,752 non-missing consensus HapMap calls

Original	Spatial normalization		
	Agree	Disagree	Missing
Agree	-	1,176 (3.6)	7,690 (23.5)
Disagree	2,518 (7.7)	7 (0.02)	2,716 (8.3)
Missing	16,887 (51.6)	1,758 (5.4)	0

Entries are frequencies (percentages) out of the 32,752 genotypes. Agreement and disagreement are with respect to the non-missing consensus HapMap calls. Separate one tailed McNemar's tests on the three pairs of discordant entries suggest significant improvement in favor of the use of spatial normalization: i.) z = 22.1 for agree versus disagree; ii.) z = 58.7 for agree versus missing; and iii.) z = 14.3 for disagree versus missing.

Another way of illustrating the effects of spatial biases is by exploiting family structure, as genotyping errors can induce Mendelian errors. We directed our attention to the genotypes obtained from the 30 parent-offspring trios of the HapMap CEU samples. Because a different cel file composition might influence genotype calling, Birdseedv2 was reapplied to this subset of 90 of the modified and original cel files. Out of all the 906,600 SNPs interrogated by the chips, the fraction of SNP trios available (i.e. no genotyping failure) for comparison based on calls from Birdseedv2 with and without spatial normalization were 99.62% and 99.50%, respectively (Table 3). The overall rates of Mendelian inconsistency were 0.534% for the standard Birdseedv2 calls and 0.525% for the Birdseedv2 calls using spatially normalized cel files. The lower rate in the latter was partly due to the conversion of SNP trios violating Mendelian inheritance into indeterminate calls or ones that follow Mendel's laws, but was best explained by the conversion of those with failed calls to calls showing no Mendelian inconsistencies (all McNemar's one-tailed test p values < 0.001 in favor of using the spatial normalization, see Additional File 5). Thus our conclusion was in line with the above, i.e. spatial bias adjustment leads to higher call rate and lower rate of genotyping error.

**Table 3:** Comparison of Birdseedv2 results on the basis of Mendelian inheritance pattern

Original	Spatial normalization		
	Consistent	Inconsistent	Missing
Consistent	26,877,547 (98.8)	2,969 (0.011)	35,887 (0.13)
Inconsistent	4,765 (0.018)	137,146 (0.50)	2,615 (0.010)
Missing	69,733 (0.26)	2,083 (0.008)	65,255 (0.24)

Entries are frequencies (percentages) of SNP trios from 90 CEU samples of Set 2 (see Method Section). Separate one tailed McNemar's tests on the three pairs of discordant entries suggest significant improvement in favor of the use of spatial normalization: i.) z = 20.4 for consistent versus inconsistent; ii.) z = 104.1 for consistent versus missing; and iii.) z = 7.75 for inconsistent versus missing.

### Set 3: 96 first-pass HapMap samples

The examples above were based on high quality hybridization chips. Here we demonstrate that the proposed methods can also be beneficial to cel files of typical quality with the use of data from the first-pass experiment. Genotypes labeled as non-redundant by the HapMap project were downloaded for comparison. After excluding SNPs not present in dbSNP and those having dissimilar allelic forms (between annotations given by Affymetrix and HapMap), a total of 899,208 SNPs were available for validation. Upon correcting for spatial biases, 261,267 genotypes of 129,053 SNPs were reassigned. As in the examples above, the proposed procedure lowered the number of failed calls of Birdseedv2 without incurring a greater degree of disagreement with results from the HapMap project (Table 4). If the commonly used 95% SNP-wise call rate threshold were to be enforced, spatial bias adjustment would have 2363 more SNPs passing the quality control.

**Table 4:** Concordance between Birdseedv2 and non-redundant HapMap calls of 261,267 genotypes

HapMap	Birdseedv2 call							
Call	Original				Spatial normalization			
	
	0	1	2	Missing	0	1	2	Missing
0	27,735 (10.6)	14,592 (5.6)	233 (0.09)	43,806 (16.8)	48,341 (18.5)	9,925 (3.8)	168 (0.06)	27,932 (10.7)
1	6,324 (2.4)	21,250 (8.1)	6,147 (2.4)	38,035 (8.1)	5,483 (2.1)	43,252 (16.6)	5,558 (2.1)	17,463 (6.7)
2	186 (0.07)	15,162 (5.8)	27,250 (10.4)	44,576 (17.1)	169 (0.06)	10,073 (3.9)	48,807 (18.7)	28,125 (10.8)
Missing	2,183 (0.84)	4,287 (1.6)	2,350 (0.90)	7,151 (2.7)	3,007 (1.2)	5,034 (1.9)	3,108 (1.2)	4,822 (1.8)

Entries are frequencies (percentages) out of the 261,267 genotypes from Set 3.

## Discussion

The quality of genotype calls will likely propagate to subsequent statistical analyses including genotype imputation [26-28], haplotype estimation [29,30], and inferences in linkage and association studies. Consequently, it is critical to further develop analytical methods to improve the measurement of SNPs. We have designed an intra-chip normalization procedure to quantify and correct for undesirable systematic intensity biases that are linked to the spatial layout of the Affymetrix SNP 6.0 array. Although we took advantage of the design of this particular chip, the proposed approach is applicable to other array platforms given the presence of randomly allocated probe replicates.

The notion of using within-array replicated features for spatial normalization is certainly not new. It has been utilized in the context of TAG [31] and cDNA [32] microarrays. To the best of our knowledge, however, this is the first study of Affymetrix SNP chips that capitalizes on such technical duplicates for the purposes of localized signal correction. The proposed procedure basically followed Yuan and Irizarry [31]. Nonetheless, their method is different from ours in two critical ways. First, in the same spirit as the use of a glog transformation, they adopted a model consisting of both additive and multiplicative bias terms so as to make neighboring 'features' comparable. The two components are estimated separately through an ad hoc subset selection of the features where the influence of other term can be regarded as negligible. However, this procedure is quite time consuming because it introduces more steps to the iteration and requires higher memory storage. Secondly, they uncovered regional biases by using a fast Fourier transform convolution. Special treatment had to be applied on the prominent defects in an effort to avoid overshadowing the less obvious artifacts. The ability of wavelets in handling sharp local structures seen in the SNP arrays is an advantage over the Fourier methods. Other smoothing techniques such as lowess and thin plate splines might also have been considered, but display drawback similar to the Fourier transform while being even less efficient computationally.

Affymetrix SNP 6.0 arrays are designed to interrogate SNPs along with genomic copy number changes. In its current form, the proposed approach only applies to SNP probes. Lack of technical replicates precludes explicit modeling of spatial effects on NP probes. Some sort of interpolation, such as a two-dimensional loess (locally weighted polynomial regression), is needed to extend the bias estimation to the entire microarray. We speculate that, because detection of copy number variation requires measurement of more subtle intensity changes, it may be even more affected by removal of spatial biases, an area that will be the focus of a future study.

## Conclusions

Our spatial normalization method operates independently on each array, making it possible to treat cel files in parallel on different processors. The usefulness of the approaches was demonstrated by the analysis of high- and typical-quality HapMap cel files. We showed that, even though only a small fraction (<1%) of all genotypes might be affected by spatial artifacts, adjusting for such biases can potentially rescue thousands of SNPs in a genetic study at the small cost of computational time. As mentioned above, the improvement in genotype call rate with equal or improved accuracy were achieved without omitting probes with outlying intensity. Exclusion of these probes might enhance the outcomes further, but this awaits further investigation.

## Authors' contributions

HSC and JPAK wrote the manuscript. HSC led the development, testing and validation of the statistical method with input and advice from TMT and KRB. Publication was authorized by JPAK. All authors have read and approved the final manuscript.

## Supplementary Material

Additional File 1 **The 'holes' filling procedure**. Additional text to describe the 'holes' filling procedure. Click here for file

Additional File 2 **Pairwise correlation matrix**. Additional text to define the pairwise correlation matrix. Click here for file

Additional File 3 **Relative gain in average R^2^**. Additional graph to show the relative gain in average R^2 ^of Set 1 over 10 iterations. Click here for file

Additional File 4 **McNemar's tests on Table 2**. Additional text to provide more details on the 3 McNemar's tests with Table 2. Click here for file

Additional File 5 **McNemar's tests on Table 3**. Additional text to provide more details on the 3 McNemar's tests with Table 3. Click here for file
