# Supplementary material for: Spatial normalization improves the quality of genotype calling for Affymetrix SNP 6.0 arrays
Source: BMC Bioinformatics. 2010 Jun 29;11:356. doi: 10.1186/1471-2105-11-356 (PMC2910027; doi:10.1186/1471-2105-11-356)
Supplement: Additional File 1 — The 'holes' filling procedure. Additional text to describe the 'holes' filling procedure. [file 1471-2105-11-356-S1.DOC]

The ‘holes’ filling procedure

- 1. identify the cells with missing deviation (*I_xy_ -* *Â_jk_*)
  2. fill these cells using the average deviation of the immediate neighbors that are not currently missing
     - most cells have 8 immediate neighbors
     - those lying at the edges have 5 immediate neighbors
     - those lying at the vertices have 3 immediate neighbors
  3. some cells remain not filled since all of their immediate neighbors have missing deviation. Repeat i and ii until all cells are filled.
