# Supplementary material for: Spatial normalization improves the quality of genotype calling for Affymetrix SNP 6.0 arrays
Source: BMC Bioinformatics. 2010 Jun 29;11:356. doi: 10.1186/1471-2105-11-356 (PMC2910027; doi:10.1186/1471-2105-11-356)
Supplement: Additional File 2 — Pairwise correlation matrix. Additional text to define the pairwise correlation matrix. [file 1471-2105-11-356-S2.DOC]

Pairwise correlation matrix

Let a_i_ = *Â_jk_* of the i-th replicated array, i = 1, …, 5. Then the pairwise correlation matrix between the m-th and n-th arrays is defined as

$\Omega_{m,n}\text{ }=cor(a_{m},a_{n})\text{ }$ , m = 1, …, 4; n = 2, …, 5; m < n.

Here, Ω_m,n_ is a 2x2 matrix. The 10 possible pairings of m and n are: (1,2), (1,3), (1,4), (1,5), (2,3), (2,4), (2,5), (3,4), (3,5) and (4,5).
