# Supplementary material for: Spatial normalization improves the quality of genotype calling for Affymetrix SNP 6.0 arrays
Source: BMC Bioinformatics. 2010 Jun 29;11:356. doi: 10.1186/1471-2105-11-356 (PMC2910027; doi:10.1186/1471-2105-11-356)
Supplement: Additional File 3 — Relative gain in average R2. Additional graph to show the relative gain in average R2 of Set 1 over 10 iterations. [file 1471-2105-11-356-S3.DOC]

Relative gain in average R^2^

Due to the large number of parameters involved (all *Ŝ_xy_* and *Â_jk_*), monitoring convergence based on absolute or relative changes of each of these parameters would be impractical. Instead the decision on the number of iteration to use was based on the relative gain in average R^2^ of our training data (i.e. Set 1).

$RI_{i}\text{ }=\text{ }\frac{\overline{R_{10}^{2}}-\overline{R_{i}^{2}}}{\overline{R_{10}^{2}}-\overline{R_{0}^{2}}}$ , i = 1, …, 10.


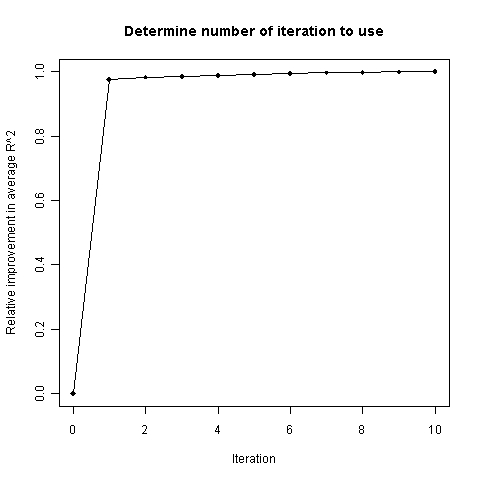


As shown in the graph, almost all of the gain in R^2^ is obtained after 1 iteration.
