# Supplementary material for: Spatial normalization improves the quality of genotype calling for Affymetrix SNP 6.0 arrays
Source: BMC Bioinformatics. 2010 Jun 29;11:356. doi: 10.1186/1471-2105-11-356 (PMC2910027; doi:10.1186/1471-2105-11-356)
Supplement: Additional File 4 — McNemar's tests on Table 2. Additional text to provide more details on the 3 McNemar's tests with Table 2. [file 1471-2105-11-356-S4.DOC]

McNemar’s tests on Table 2

| Original | Normalized | | |
| --- | --- | --- | --- |
|  | Agree^*^ | Disagree^*^ | Missing |
| Agree^*^ | - | AD_1_ = 1176 | AM_1_ = 7690 |
| Disagree^*^ | AD_2_ = 2518 | 7 | DM_1_ = 2716 |
| Missing | AM_2_ = 16887 | DM_2_ = 1758 | 0 |

^*^with HapMap call

1. Agree vs. disagree

We would like to have more calls to change from disagree to agree rather than the reverse.

H_0_: AD_1_ = AD_2_; H_A_: AD_1_ < AD_2_

$z=\frac{AD_{2}-AD_{1}-0.5}{\sqrt{AD_{2}+AD_{1}}}=22.1$, which is highly significant.

1. Agree vs. missing

We would like to have more calls to change from missing to agree rather than the reverse.

H_0_: AM_1_ = AM_2_; H_A_: AM_1_ < AM_2_

$z=\frac{AM_{2}-AM_{1}-0.5}{\sqrt{AM_{2}+AM_{1}}}=58.7$, which is highly significant.

1. Disagree vs. missing

We would like to have more calls to change from disagree to missing rather than the reverse.

H_0_: DM_1_ = DM_2_; H_A_: DM_1_ > DM_2_

$z=\frac{DM_{1}-DM_{2}-0.5}{\sqrt{DM_{1}+DM_{2}}}=14.3$, which is highly significant.

All three tests suggest significantly better results in favor of using the normalized cel files.
