# Supplementary material for: Spatial normalization improves the quality of genotype calling for Affymetrix SNP 6.0 arrays
Source: BMC Bioinformatics. 2010 Jun 29;11:356. doi: 10.1186/1471-2105-11-356 (PMC2910027; doi:10.1186/1471-2105-11-356)
Supplement: Additional File 5 — McNemar's tests on Table 3. Additional text to provide more details on the 3 McNemar's tests with Table 3. [file 1471-2105-11-356-S5.DOC]

McNemar’s tests on Table 3

| Original | Spatial normalization | | |
| --- | --- | --- | --- |
|  | Consistent | Inconsistent | Missing |
| Consistent | 26,877,547 | CI_1_ = 2,969 | CM_1_ = 35,887 |
| Inconsistent | CI_2_ = 4,765 | 137,146 | IM_1_ = 2,615 |
| Missing | CM_2_ = 69,733 | IM_2_ = 2,083 | 65,255 |

1. Consistent vs. inconsistent

We would like to have more SNP trios to change from inconsistent to consistent rather than the reverse.

H_0_: CI_1_ = CI_2_; H_A_: CI_1_ < CI_2_

$z=\frac{CI_{2}-CI_{1}-0.5}{\sqrt{CI_{2}+CI_{1}}}=20.4$, which is highly significant.

1. Consistent vs. missing

We would like to have more SNP trios to change from missing to consistent rather than the reverse.

H_0_: CM_1_ = CM_2_; H_A_: CM_1_ < CM_2_

$z=\frac{CM_{2}-CM_{1}-0.5}{\sqrt{CM_{2}+CM_{1}}}=104.1$, which is highly significant.

1. Inconsistent vs. missing

We would like to have more SNP trios to change from inconsistent to missing rather than the reverse.

H_0_: IM_1_ = IM_2_; H_A_: IM_1_ > IM_2_

$z=\frac{IM_{1}-IM_{2}-0.5}{\sqrt{IM_{1}+IM_{2}}}=7.75$, which is highly significant.

All three tests suggest significantly better results in favor of using the normalized cel files.
